# Insights into Regulation of the miR-17-92 Cluster of miRNAs in Cancer

**DOI:** 10.3389/fmed.2015.00064

**Published:** 2015-09-08

**Authors:** Cesar Seigi Fuziwara, Edna Teruko Kimura

**Affiliations:** ^1^Department of Cell and Developmental Biology, Institute of Biomedical Sciences, University of São Paulo, São Paulo, Brazil

**Keywords:** microRNA, miR-17-92, cancer, transcription, processing

## Abstract

Overexpression of the miR-17-92 cluster is a key oncogenic event in various cancer types. The oncogenic effect of the miR-17-92 cluster is enhanced by cooperation between its members in targeting tumor-suppressive proteins and pathways such as PTEN and TGFβ signaling. However, in the case of miR-19a and miR-19b, these have been shown to have a preponderant role in the cluster’s oncogenicity. Important studies have revealed the influence of the Myc proto-oncogene family in the transcriptional regulation of miR-17-92. Recent findings show that other oncogenic signaling pathways, such as those of Notch and Sonic Hedgehog, activate miR-17-92 in cancer. Notwithstanding, another layer of complexity has been added by the influence of the relevant primary miR-17-92 tertiary structure during processing to mature miRNA. In this review, we attempt to integrate current transcriptional and post-transcriptional knowledge to enhance our global understanding of the coordinated up-regulation of miR-17-92 in cancer.

## Introduction

MicroRNAs (miRNAs), a class of small non-coding RNAs (~22 nucleotides), negatively regulate protein translation of target mRNAs post-transcriptionally to fine-tune the control of cell homeostasis. However, the unbalanced expression of miRNA is a hallmark of several diseases, including cancer (1). In this regard, “oncomiR” refers to a miRNA that induces or potentiates oncogenic signaling when its expression is deregulated in cancer. Some oncomiRs, *per se*, have a substantial influence on oncogenic processes, such as miR-17-92 (oncomiR-1).

A plethora of important studies corroborate a major oncogenic role for the miR-17-92 cluster as a driver or secondary event that enhances the oncogenic process in different cancer types (2). Therefore, in this mini review, we intend to focus on several novel findings concerning the biological role of miR-17-92 and new molecular aspects of miR-17-92 transcriptional and post-transcriptional regulation.

## Biological Role of the miR-17-92 Cluster

The miR-17-92 cluster (oncomiR-1) is located in an intron of *MIR17HG* [miR-17-92 cluster host gene (non-protein coding)] on chromosome 13 (13q31.3) (3). The primary transcript of miR-17-92 (pri-miR-17-92) is a ~0.8-kb-long polycistron that is processed into seven different mature miRNAs: miR-17 (miR-17-5p and miR-17-3p), miR-18a, miR-19a, miR-19b, miR-20a, and miR-92a. This genomic locus, previously described as *C13orf25*, is amplified in hematopoietic malignancies (13q31–q32 amplification) (4), with overexpression of miR-17-92 observed in several types of cancer, including lung, breast, colon, pancreas, prostate, thyroid, and lymphoma (3, 5–8).

The artificial manipulation of miR-17-92 expression in transgenic mice showed that the cluster is essential to normal mouse development (9, 10), with the correct timing of miR-17-92 activation essential to lung development (9). A high abundance of miR-17-5p, miR-18, miR-19b, miR-20a, and miR-92 was observed at the E11.5 stage of mouse development, while at E17.5, miR-17-92 levels were reduced. In studies of transgenic mice where miR-17-92 overexpression was targeted to lung epithelium using the surfactant protein C promoter, the authors observed abnormal lung development, consisting of very few normal alveoli and with obvious epithelia hyperplasia, and mice dying soon after birth (9). Indeed, high levels of miR-17-92 expression are observed in aggressive lung cancer (5), pointing to a role for the cluster in lung cancer oncogenesis.

The generation of knock-outs of miR-17-92 (miR-17-92^Δneo/Δneo^) in newborn mice resulted in death soon after birth (10). The cause of the observed post-natal lethality was severe hypoplastic lungs and ventricular septal defects. In humans, the homozygous germline deletion of *MIR17HG* significantly reduced mature miR-17-92 levels and is associated with a syndrome characterized by microcephaly, short stature, and digital defects (11). These developmental abnormalities were recapitulated in transgenic mice with a targeted deletion of miR-17-92.

## Oncogenic Role of the miR-17-92 Cluster

**Figure 1:**
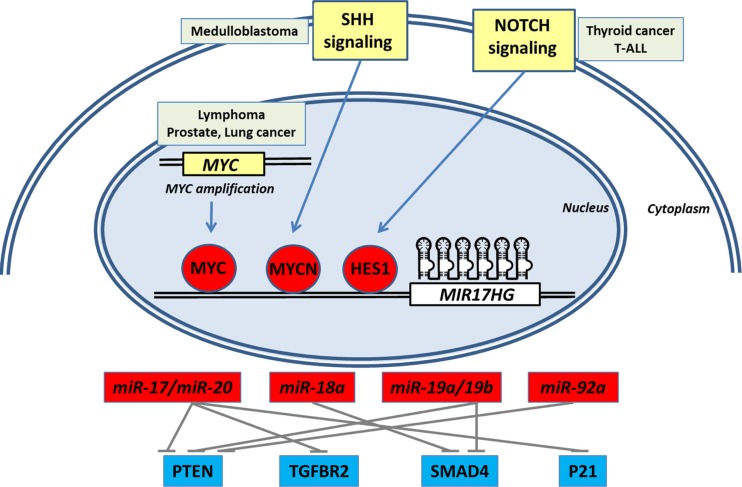
**Coordinated transcriptional activation of miR-17-92 by oncogenic signaling pathways synergistically down-regulates important negative regulators of cell growth and proliferation signaling in cancer**.

The integration of different datasets from The Cancer Genome Atlas, available from the online cBioportal for Cancer Genomics website, does not show major genetic alterations in *MIR17HG* in different types of cancer, despite a few cases of genomic amplification (below 10% in some cancers) (12). This indicates that transcriptional (Figure 1) and post-transcriptional processes of miR-17-92 are the key in regulating mature miRNA levels.

An initial report that miR-17-92 contributed to B-cell lymphomagenesis in *Eu-myc* transgenic mice pointed to an oncogenic role for the cluster (3). In this model, lymphoma is driven by c-Myc oncogene overexpression controlled by an immunoglobulin heavy-chain enhancer (*Eu-myc*) in transgenic mice, and overexpression of the miR-17-92 cluster accelerates the development of c-Myc-induced B-cell lymphoma.

Two independent studies have shown a causative oncogenic role for miR-17-92 in lymphomagenesis as a bona fide oncogene (13, 14). In the first, the targeted expression of miR-17-92 to B cells (Eμ-*miR-17-92* mice) led to the development of B-cell malignancy (three classes of B-cell lymphoma or leukemia) with high penetrance (~80%) and massive spleen enlargement (14). In the second study, targeted miR-17-92 expression to B cells (miR-17-92 Tg/Tg; CD19 Cre) also induced B-cell lymphoma development, followed by PTEN down-regulation and enhancement of the mTOR pathway (13), altogether, showing a potent oncogenic role for miR-17-92 *per se*.

Several lines of evidence reveal the importance of balancing the expression of mature miRNAs to elicit oncogenic effects. Two independent studies dissected the contribution of singular miR-17-92 components to reveal that miR-19a and miR-19b are the key oncogenic miRNAs in the cluster (15, 16). Firstly, Olive et al. showed enhanced lethality of irradiated mice when injected with Eμ-*myc* hematopoietic stem and progenitor cells selectively transduced with miR-19b (16). Moreover, a specific mutation to disrupt the hairpin stem of miR-19a and miR-19b, and consequently the biogenesis of these mature miRNAs, caused a delay in the onset of B-cell lymphoma and reduced animal deaths. Importantly, in the second study, Mu et al. showed that deletion of the entire miR-17-92 reduced B-cell lymphoma proliferation in *Eu-myc* transgenic mice (15). Moreover, selective overexpression of miR-19a and miR-19b in *Eu-myc* transgenic mice rescued the growth advantage of lymphoma cells.

More recently, Han et al. showed the essential role of miR-19 in prostate cancer tumorigenesis in mice presenting high levels of c-Myc (Hi-Myc; miR-17-92^+/+^) targeted to prostate cells. In these animals, at the age of 10 months, invasive prostate cancer is detected in all animals, whereas no invasive tumor is observed in the miR-19 deleted mice (Hi-Myc; miR-17-92^Δ19/Δ19^) (17).

Disruption of the miR-19:miR-92 ratio to enhance miR-19 over miR-92 levels is observed in pre-malignant and malignant *Eu-myc* B cells compared with normal B cells (18). Moreover, the molecular manipulation of miR-92 levels to overcome miR-19 in *Eu-myc* B cells induced apoptosis by caspase activation. In thyroid cancer, induction of the *BRAF* oncogene leads to overexpression of miR-17-92 cluster components, with a clear shift of miR-19a/b levels to overcome miR-92a. Interestingly, the protective effect of high iodine treatment was observed by blocking an miR-19 increase while miR-92 levels remained the same (19). These studies reinforce evidence for miR-19 as the oncogenic miRNA and for miR-92 as a negative regulator of the cluster.

## Transcriptional Regulation and Processing of miR-17-92

Two major mechanisms are involved in the regulation of mature miR-17-92 levels: transcriptional, which implicate promoter activation/repression, and post-transcriptional, which concern predominantly primary miRNA processing. In this regard, not only does the classical importance of oncogenic pathways emerge but also the role of pri-miRNA tertiary structure processing and the action of RNA-binding proteins.

To understand the transcriptional regulation of miR-17-92, it is imperative to analyze its putative promoter region. One of the best characterized regulators of miR-17-92 transcription is the proto-oncogene, c-Myc, which is amplified in different types of tumors (12). The miR-17-92 putative promoter region in both humans and rodents presents consensus-binding sites for c-Myc. The overexpression of c-Myc induces its binding to the miR-17-92 promoter and activates cluster transcription in HeLa cells (20), whereas the knock-down of *c-Myc* drastically reduces miR-17-92 levels in the same cell line (21).

The expression of miR-17-92 is also induced by another member of the *Myc* family, N-myc, a target of the activation of the Sonic Hedgehog (SHH)/Patched signaling pathway in medulloblastoma (22). Overexpression of N-myc, or the treatment of cerebellar granule neuron precursors with exogenous Shh, induces miR-17-92 *in vitro*, and also medulloblastoma derived from Patched^+/−^ or SmoA1 mice shows overexpression of miR-17-92 (22). Among miRNA cluster members, miR-19 is the highest expressed in SHH-derived medulloblastoma (Patched^+/−^) and the treatment with anti-miR-19 intravenously inhibited xenograft tumor growth (23). Indeed, miR-17-92 is required for medulloblastoma formation as the cluster deletion in a transgenic Patched^+/−^ mouse model blocked tumor development (24). Altogether, these studies indicate an important role for the Myc family in the transcription of the miR-17-92 cluster.

Regardless of the classical influence of c-Myc upon miR-17-92 transcription, Notch signaling plays an important role in the regulation of miR-17-92 levels. The putative miR-17-92 promoter presents predicted binding sites for HES1, a Notch signaling effector. Notch signaling is overexpressed in thyroid cancer (25) and *NOTCH1* knock-down reduces miR-19 levels in papillary thyroid cancer (19). The activation of Notch intracellular domain (NICD) induces substantial miR-17-92 expression in normal thyroid follicular cells, while BRAF^V600E^ oncogene induction also increases miR-17-92 via Notch signaling activation.

Cooperation between miR-19 and Notch1 activation was also observed in T-cell acute lymphoblastic leukemia (T-ALL) (26). In this study, *NOTCH1* gene translocation t(9;14)(q34;q11) was found concomitant with the miR-17-92 cluster t(13;14)(q32;q11) in one leukemic clone. Moreover, the transduction of hematopoietic progenitor cells to overexpress the NICD, and posterior implantation into irradiated receptor mice, caused T-ALL in 20% of animals, while the concomitant transduction of NICD together with miR-19 lead to 100% of animals developing T-ALL after 2 months. The high frequency of Notch1-activating mutations in T-ALL, occurring in more than 50% of clinical cases (27), and the higher expression of miR-19 in these patients (28) highlight the important cooperation between Notch signaling and miR-17-92 in T-ALL.

Although transcribed as a single primary transcript, pri-miR-17-92 processing produces distinct singular mature miRNA levels, indicating the existence of a differential processing mechanism. Recent studies elegantly showed that the molecular structure of pri-miR-17-92 at secondary and tertiary levels conferred intrinsic characteristics that influenced miRNA processing.

Pri-miR-17-92 is transcribed as a long ~800 nt RNA that folds into a characteristic globular tertiary structure, which enables differential processing to yield mature miRNAs (29). The molecular structure of the folded RNA presents regions that are solvent accessible or inaccessible, culminating in differential enzymatic access and processing. Indeed, the Drosha enzyme’s access to the 3′-core domain of miRNAs (miR-19b and miR-92a) is impaired due to such globular folding. Moreover, deletion of the 5′-region of the cluster abrogates this protection, as does deletion of the miR-19 precursor itself or an non-miRNA containing stem-loop (NMSL) sequence, resulting in the processing of miR-92a (29). The NMSL region is located between miR-19b and miR-92a, and forms tertiary contacts with pre-miR-19b that represses Drosha processing of pre-miR-92a, and contributes to the maintenance of pri-miR-17-92 globular structure (30). Moreover, biochemical properties of pri-miR-17-92’s three-dimensional structure confer a kinetic barrier to its processing and influences protein recognition sites (31).

Nevertheless, these studies do not consider the possible existence of an interaction between RNA-binding proteins and the pri-miR-17-92 cluster to modulate its processing. Indeed, hnRNP A1 interacts with miR-17-92 and facilitates the processing of pre-miR-18 (32). Therefore, the existence of an as yet to be characterized mechanism, whereby RNA-binding proteins selectively enhance or blunt miR-17-92 components, could contribute to miR-17-92’s fine-tuned expression and tissue specificity.

## Targeting of Tumor-Suppressive Pathways in Cancer by miR-17-92

The target prediction of miR-17-92 components, based on seed sequence homology, divides the miRNAs into four families: miR-17 (miR-17 and 20), miR-18, miR-19 (miR-19a and 19b), and miR-92 families. A single, predicted target mRNA is frequently observed to harbor multiple binding sites for miR-17-92 components in the 3′-UTR, indicating a synergistic mechanism of action.

An important target of the miR-17-92 cluster is the tumor suppressor *PTEN* (Table 1) (6, 15, 16). The existence of multiple conserved binding sites for miR-19a/b, miR-17, and miR-20a in the 3′-UTR of *PTEN* mRNA highlights the cooperative effect of miR-17-92 cluster components in blunting key mRNAs to enhance oncogenesis. The down-regulation of PTEN levels is a key step during tumorigenesis and potentiates the activation of AKT/mTOR growth and survival signaling. Indeed, overexpression of miR-19 causes a strong inhibition of PTEN levels and the enhancement of pAkt. Also, an important increase in the phosphorylation of the S6 ribosomal protein is observed, indicative of the activation of mTOR signaling (16). *In vivo*, the deletion of miR-17-92 in *Eu-myc* B-cells results in a lymphoma showing increased levels of PTEN in nude mice (15).

**Table 1:** **Validated miR-17-92 targets**.

Validated targets	miR-17-92 cluster						Reference
	*miR-17*	*miR-18a*	*miR-19a*	*miR-19b*	*miR-20a*	*miR-92a*	
*PTEN*	x		x	x	x	x	(6, 15, 16)
*SMAD4*		x	x	x			(19, 33, 34)
*TGFBR2*	x				x		(33, 34)
*CDKN1A (P21)*	x				x		(35)

Another important tumor-suppressive pathway targeted by several members of miR-17-92 is the TGFβ signaling pathway (36), reinforcing the synergistic role of cluster miRNAs (Table 1). The TGFβ signaling pathway is an important regulator of epithelial cell proliferation, triggering an antiproliferative signal mediated by p21. However, miR-17-92-mediated blunting of signal transduction by TGFβ, by down-regulating its signaling components, would enhance cell proliferation and make cancer cells refractory to TGFβ1 signaling.

Recent studies have confirmed that *TGFBR2* is targeted by miR-17/20, while *SMAD2/SMAD4* is regulated by miR-18 in neuroblastoma cells (33). Moreover, *Smad4* is also a target of miR-19a/miR-19b in thyroid follicular cells (19). Indeed, in thyroid cancer, *BRAF*-mediated miR-17-92 up-regulation leads to a reduction of Smad4 and Tgfbr2 proteins and a loss of responsiveness to TGFβ1. The blockage of miR-19a/b with anti-miR restores TGFβ1 G1 cell cycle arrest in thyroid cancer cells. In glioblastoma cells, activation of miR-17-92 blunted TGFβ-induced gene expression signature (34). In addition, miR-20 targets *CDKN1A* (P21), an important negative regulator of cell cycle progression activated by TGFβ in colorectal cancer cells and blocks TGFβ-induced antiproliferative effect (35).

## Future Directions

To functionally understand the biogenesis of mature miR-17-92 miRNAs, it is necessary to integrate transcriptional and post-transcriptional regulatory mechanisms.

*Myc* family genes exert important transcriptional effects on miR-17-92; however, a considerable fraction of cancers do not show genetic alterations in these proto-oncogenes. In this regard, the activation of oncogenic signaling, such as Shh and Notch, is involved in the transcriptional activation of miR-17-92. Nevertheless, many other predicted oncogenic transcription factors may bind to, and regulate, the miR-17-92 promoter.

Furthermore, after primary miR-17-92 transcription, its post-transcriptional regulation is responsible for fine-tuning the generation of miR-17-92 miRNAs. The molecular tertiary structure of the miR-17-92 primary transcript emerges as an important modulator of miRNA processing machinery, but does not fully explain the distinct patterns in expression of miR-17-92 components as observed experimentally. Therefore, RNA-binding proteins may play a role in selectively targeting and regulating miRNAs of the cluster during processing.

Indeed, understanding the biological role of miR-17-92 cluster is essential for translating bench knowledge to the bedside. *In vitro* studies revealed antitumorigenic effects of targeting miR-17-92 in cancer cell lines. Also, *in vivo* animal models have shed light into the potentiality of targeting miR-17-92 components therapeutically. The use of intravenous delivery of anti-miR-17-92 for the treatment of allograft medulloblastoma tumor in immune-compromised mice resulted in blockage of tumor growth (23), indicating miR-17-92 as a feasible therapeutic target. However, some issues regarding selective anti-miR delivery to cancer cells and its side effects in animal models still need to be addressed by further studies in order to permit the safe application of anti-miR-17-92 as a therapeutic adjuvant for cancer treatment.

## Conflict of Interest Statement

The authors declare that the research was conducted in the absence of any commercial or financial relationships that could be construed as a potential conflict of interest.
